# Enhancing voriconazole therapy in liver dysfunction: exploring administration schemes and predictive factors for trough concentration and efficacy

**DOI:** 10.3389/fphar.2023.1323755

**Published:** 2024-01-04

**Authors:** Yichang Zhao, Huaiyuan Liu, Chenlin Xiao, Jingjing Hou, Bikui Zhang, Jiakai Li, Min Zhang, Yongfang Jiang, Indy Sandaradura, Xuansheng Ding, Miao Yan

**Affiliations:** ^1^ Department of Clinical Pharmacy, The Second Xiangya Hospital of Central South University, Changsha, China; ^2^ Institute of Clinical Pharmacy, Central South University, Changsha, China; ^3^ School of Basic Medicine and Clinical Pharmacy, China Pharmaceutical University, Nanjing, China; ^4^ Department of Infectious Disease, The Second Xiangya Hospital of Central South University, Changsha, China; ^5^ School of Medicine, University of Sydney, Sydney, NSW, Australia; ^6^ Centre for Infectious Diseases and Microbiology, Westmead Hospital, Sydney, NSW, Australia

**Keywords:** voriconazole, liver dysfunction, trough concentration, efficacy, therapeutic drug monitoring

## Abstract

**Introduction:** The application of voriconazole in patients with liver dysfunction lacks pharmacokinetic data. In previous study, we proposed to develop voriconazole dosing regimens for these patients according to their total bilirubin, but the regimens are based on Monte Carlo simulation and has not been further verified in clinical practice. Besides, there are few reported factors that significantly affect the efficacy of voriconazole.

**Methods:** We collected the information of patients with liver dysfunction hospitalized in our hospital from January 2018 to May 2022 retrospectively, including their baseline information and laboratory data. We mainly evaluated the efficacy of voriconazole and the target attainment of voriconazole trough concentration.

**Results:** A total of 157 patients with liver dysfunction were included, from whom 145 initial and 139 final voriconazole trough concentrations were measured. 60.5% (95/157) of patients experienced the adjustment of dose or frequency. The initial voriconazole trough concentrations were significantly higher than the final (mean, 4.47 *versus* 3.90 μg/mL, *p* = 0.0297). Furthermore, daily dose, direct bilirubin, lymphocyte counts and percentage, platelet, blood urea nitrogen and creatinine seven covariates were identified as the factors significantly affect the voriconazole trough concentration. Binary logistic regression analysis revealed that the lymphocyte percentage significantly affected the efficacy of voriconazole (OR 1.138, 95% CI 1.016–1.273), which was further validated by the receiver operating characteristic curve.

**Conclusion:** The significant variation in voriconazole trough concentrations observed in patients with liver dysfunction necessitates caution when prescribing this drug. Clinicians should consider the identified factors, particularly lymphocyte percentage, when dosing voriconazole in this population.

## 1 Introduction

Patients with liver dysfunction are at a heightened risk of invasive fungal infections (IFI) due to immune dysfunction, frequent hospitalization, and altered intestinal microflora (Pijls et al., 2013; Hassan et al., 2014; Lahmer et al., 2022). The mortality of patients with liver dysfunction who develop IFI remains high (Verma et al., 2019), necessitating the use of antifungal drugs.

Voriconazole (VRZ), a broad-spectrum second-generation triazole antifungal drug, is recommended for preventing or treating IFI by both Chinese guideline and the Infectious Diseases Society of America (Patterson et al., 2016; Chen et al., 2018). Its trough concentration was relevant to the efficacy and safety (Johnson et al., 2010; Lin et al., 2022). Conducted a systematic meta-analysis encompassing 21 studies involving 1158 patients. The findings indicated that a voriconazole level of 0.5 mg/L should be regarded as the lower threshold associated with efficacy. Furthermore, a trough concentration exceeding 3.0 mg/L is linked to an augmented risk of hepatotoxicity, particularly for the Asian population, while a concentration exceeding 4.0 mg/L is associated with an increased risk of neurotoxicity (Hanai et al., 2021). Therefore, voriconazole exposure is an important factor affecting its safety and efficacy. Maintaining VRZ trough concentrations within the therapeutic target range could achieve maximal antifungal efficacy whilst minimizing the incidence of serious concentration-dependent adverse effects.

The intra-individual and inter-individual variation in VRZ concentrations in patients with liver dysfunction is considerable (Lin et al., 2020). VRZ product information recommended that in patients with Child-Pugh class A and B cirrhosis (CP-A and CP-B), the loading dose remain unchanged whilst the maintenance dose should be reduced by half. Unfortunately, there are no recommendations for patients with Child-Pugh class C cirrhosis (CP-C) due to lack of pharmacokinetic data. Hence currently VRZ dosing in CP-C patients in clinical practice is mainly determined by the physicians’ experience. Previous studies (Wang et al., 2018a; Wang et al., 2018b) suggested that optimal dosing of VRZ in patients with liver dysfunction required further study.

Multiple studies (Wang et al., 2014; Chen et al., 2015) have shown that biochemical liver function markers were key covariates of voriconazole pharmacokinetic parameters. Our previous study (Tang et al., 2021) proposed adjusting VRZ dose according to total bilirubin (TBIL) in patients with liver dysfunction to increase target attainment, but the regimens still require clinical validation. Therefore, further research is needed to analyze the rationality of optimal dose regimens for VRZ therapy in patients with liver dysfunction. Concurrently, considering the unpredictable VRZ concentrations and efficacy, the study also aimed to explore key factors affecting its concentration and the efficacy.

## 2 Materials and methods

### 2.1 Patients

Patients hospitalized in the Department of Infectious Diseases, the Second Xiangya Hospital of Central South University from January 2018 to May 2022 were eligible for inclusion if they met the following criteria: 1) diagnosed with liver dysfunction, such as liver cirrhosis, acute-on-chronic liver failure; 2) prescribed VRZ to treat or prevent IFI; 3) VRZ trough concentration was measured at least once. Exclusion criteria included: 1) pregnancy or lactation; 2) using rifampicin, isoniazid, phenytoin and potent CYP450 inducers or inhibitors during VRZ therapy; 3) poor patient compliance. The study was approved by the ethics committee of the Second Xiangya Hospital of Central South University, approved number: [2022 Ethical Review CR No. (066)], and the study was performed in accordance with the Helsinki Declaration of 1964, and its later amendments.

### 2.2 Data collection

Demographic information, laboratory data, and other clinical data were collected from the hospital information system (HIS), including age, gender, body weight, dosing regimen, VRZ trough concentrations, efficacy, renal and liver function, and routine pathology tests. The model for end-stage liver disease (MELD) score (Kamath and Kim, 2007) and Child-Pugh class (Tsoris, 2022) were calculated by the corresponding formula.

### 2.3 Dose

The dose of VRZ was determined by the physicians. CP-A and CP-B patients received either the VRZ instruction dose or TBIL-based dosing regimens. The dosing regimens of CP-C patients were determined by the physicians. The details of these dosing regimens are shown below. VRZ instruction dosing regimens: for CP-A and CP-B patients, loading dose 400 mg every 12 h (q12h), maintenance dose 100 mg q12h. As for CP-C patients, there are no recommendations due to lack of pharmacokinetic data, and the dosing regimens were determined by the physicians. The TBIL-based dosing regimens are as follows (Tang et al., 2021): 51 μmol/L < TBIL, loading dose 400 mg q12h, maintenance dose 100 mg q12h. 51 μmol/L ≤ TBIL<171 μmol/L, loading dose 200 mg q12h, maintenance dose 100 mg per day. TBIL≥171 μmol/L, loading dose 200 mg q12h, maintenance dose 50 mg per day. The therapeutic target range of VRZ is 1.0–5.5 μg/mL.

### 2.4 Efficacy

According to previous studies (Pascual et al., 2008; Yang et al., 2021) and the situation of our hospital, we developed the criteria of VRZ efficacy evaluation. The response of IFI was assessed by 2008 EORTC/MSG (Pascual et al., 2008) and the 2020 EORTC/MSGERC (Donnelly et al., 2020) definitions. The evaluation was comprehensive and involved clinical manifestations, G/GM tests, and radiology. Additionally, we adhered to the 2008 EORTC/MSG criteria for coding IFDs and outcomes. A favorable response includes ‘complete response’ or ‘partial response’, while ‘stable disease’, progression, or death due to the infection are classified as failures. For prophylaxis and empirical therapy, success is defined as the completion of therapy without recurrent or breakthrough fungal infections, no discontinuation due to AEs, and survival at the end of therapy (Pieper et al., 2012).

### 2.5 Measurement of VRZ trough concentrations and CYP2C19 genotype

The VRZ trough concentrations were measured using the automatic two-dimensional high-performance liquid chromatography system (Demeter Instrument Co. Ltd., Changsha, China) (Zhao et al., 2021a; Zhao et al., 2021b). Specifically, the calibration range for Voriconazole plasma concentrations is 0.24–12.04 mg/L. The intra-day and inter-day precisions were 1.94%–2.22% and 2.15%–6.78%, respectively. The absolute and relative recovery ranged from 88.2% to 93.6% and 94.2%–105.3%. The stability of the blood sample at room temperature for 8 h and at −20°C of 3 repeated freeze-thaw cycles was within ±8% and ± 10%, respectively. The laboratory participated in the annual national external quality assessment scheme. Trough concentration was defined in accordance with our previous study (Zhao et al., 2021a). Additionally, the initial VRZ trough concentration was determined as the trough concentration following the first administration, while the final VRZ trough concentration was defined as the last measured trough concentration or the trough concentration after the last dose adjustment.

Blood samples were obtained to determine the CYP2C19 genotype. The DNA was extracted and purified by using the EZNA^®^ SQ Blood DNA Kit Ⅱ (Omega BioTek, Ink., Norcross, GA, USA), followed by genotyping using the Sanger dideoxy DNA sequencing method with the ABI3730xl fully automatic DNA Analyzer (ABI Co., Biosune Biotechnology Co., Ltd., Shanghai, China). Based on genotype, the CYP2C19 phenotypes were classified into five categories (Moriyama et al., 2017): ultrarapid metabolizers (UM): CYP2C19*17*17, rapid metabolizers (RM): CYP2C19*1*17, normal metabolizers (NM): CYP2C19*1*1, intermediate metabolizers (IM): CYP2C19*1*2, *1*3, *2*17 and *3*17, poor metabolizers (PM): CYP2C19*2*2, *2*3 and *3*3.

### 2.6 Statistical analysis

The statistical analysis was performed using SPSS version 25.0. Comparisons of quantitative data were made using Student’s t-test and one-way ANOVA or Wilcoxon signed-rank test and Mann-Whitney *U* test, according to its distribution. Categorical data was analyzed using the χ^2^ test or Fisher’s exact test. Spearman correlation analysis was performed to evaluate the relationship between covariates and efficacy. The stepwise method was used to construct the multiple linear regression model of the covariates that affecting VRZ trough concentration. We used a *p*-value threshold of 0.05 for variable inclusion, considering covariates with *p*-values less than 0.05 as statistically significant and including them in the model. Conversely, we set a removal threshold at a *p*-value of 0.1, removing covariates with *p*-values exceeding this threshold at each step of the process. This process continued iteratively until no more variables met the inclusion or removal criteria. These *p*-value thresholds were applied systematically to identify the most relevant covariates influencing VRZ trough concentration. Meanwhile, a variance inflation factor (VIF) < 5 was considered indicative of non-multicollinearity. And the logistic regression was performed to analyze the relationship between the screened covariates and the efficacy. The accuracy of screened covariates in predicting efficacy were assessed using receiver operating characteristic (ROC) curve analysis. A *p*-value of <0.05 was considered statistically significant. Graphpad Prism version 9.0.0 was used to visualize the data.

## 3 Results

### 3.1 Patient characteristics

157 patients were enrolled in this study, their demographics were summarized in Table 1. Among the participants, CYP2C19 genotypes were detected in 130 patients, and their characteristics are presented in Table 1. The pharmacogenomic profiles of CYP2C19 were PM (10%), IM (44.6%), NM (44.6%), and RM (0.8%). Notably, no patients with UM metabolic type were included in our study. In terms of patients whose Child-Pugh class and MELD score were calculable (n = 153 and 112, respectively), the majority of Child-Pugh class was CP-C, and the mean MELD score was 23.6. Additionally, Table 2 outlines the baseline biochemical parameters of the study cohort.

**TABLE 1:** Demographic information of the study cohort.

Characteristics	Value^a^
Sex (male), N (%)	133 (84.7%)
Age (year)^b^	48.73 ± 12.438
Body weight (kg)	62.0 (55.0–70.0)
CYP2C19 genotypes, N (%)	
*1*1	58 (44.6%)
*1*2	45 (34.6%)
*1*3	10 (7.7%)
*1*17	1 (0.8%)
*2*2	11 (8.5%)
*2*3	2 (1.5%)
*2*17	2 (1.5%)
*3*17	1 (0.8%)
Child-Pugh Class, N (%)	
A	1 (0.7%)
B	28 (18.3%)
C	124 (81.0%)
MELD Score^b^	23.6 ± 9.9

^a^ Continuous covariates are described as mean ± SD, or median (IQR) according to the Gaussian distribution.

^b^ Shows that the variable is Gaussian distribution according to Shapiro-Wilk test.

**TABLE 2:** Baseline biochemical parameters of the study cohort.

Parameters	Value^a^
RBC (×10^9^/L)	2.81 (2.35–3.05)
WBC (×10^9^/L)	6.76 (3.60–7.53)
PLT (×10^9^/L)	79 (43–97)
HGB (g/L)	91 (79–103)
LYM^b^ (×10^9^/L)	0.96 (0.63–1.15)
LYM% (%)	17.5 (11.0–22.6)
NEUT^b^ (×10^9^/L)	5.32 (2.67–5.63)
NEUT% (%)	71.5 (65.5–80.8)
ALT (U/L)	63.88 (28.75–73.40)
AST (U/L)	111.84 (50.85–137.65)
TBIL (μmol/L)	313.0 (161.5–438.2)
DBIL (μmol/L)	222.5 (114.0–327.5)
ALB (g/L)^b^	32.24 ± 4.99
ALP (U/L)^b^	137.50 ± 56.80
GGT (U/L)	91.35 (42.95–118.70)
CRP (mg/L)	37.86 (11.95–58.68)
PCT (μg/L)	2.4300 (0.5900–2.4000)
BUN (mmol/L)	10.38 (4.22–11.81)
CREA (μmol/L)	106.4 (63.8–122.8)
UA (μmol/L)	233.2 (126.7–301.0)
INR	2.32 (1.60–2.81)
PT (second)	25.5 (19.2–29.8)
PT%^b^	44.6 ± 13.8

RBC, red blood cell; WBC, white blood cell; PLT, platelet; HGB, hemoglobin; LYM#, lymphocyte counts; LYM%, lymphocyte percentage; NEUT#, neutrophils counts; NEUT%, neutrophils percentage; ALT, alanine aminotransferase; AST, aspartate aminotransferase; TBIL, total bilirubin; DBIL, direct bilirubin; ALB, albumin; ALP, alkaline phosphatase; GGT, glutamyl transferase; CRP, C-reactive protein; PCT, procalcitonin; BUN, blood urea nitrogen; CREA, creatinine; UA, uric acid; INR, international normalized ratio; PT, prothrombin time; PT%, prothrombin activity.

^a^ Continuous covariates are described as mean ± SD, or median (IQR) according to the Gaussian distribution.

^b^ Shows that the variable is Gaussian distribution according to Shapiro-Wilk test.

### 3.2 Concentrations of VRZ

**FIGURE 1:**
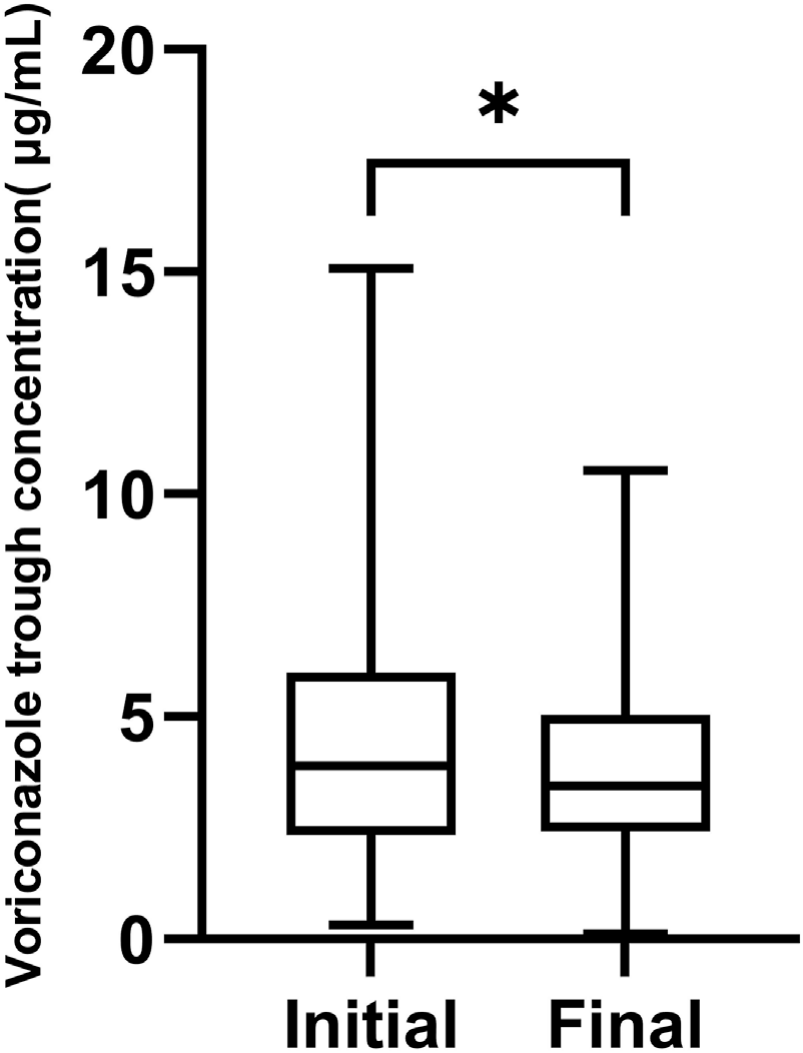
The box chart of initial and final voriconazole trough concentrations,*p < 0.05.

604 VRZ plasma concentrations from 157 patients were measured during the study. Of these concentrations, 145 were initial trough concentrations and 139 were final trough concentrations. The mean initial and final VRZ trough concentrations were 4.47 μg/mL (range 0.32–15.07 μg/mL) and 3.90 μg/mL (range 0.12–10.65 μg/mL) respectively. The final VRZ trough concentrations were significantly lower than the initial (*p* = 0.0297, Figure 1).

Of the 145 initial VRZ trough concentrations, 101 (69.2%) achieved the therapeutic target range of 1.0–5.5 μg/mL, while 4 were below 1.0 μg/mL and 40 were above 5.5 μg/mL. Of the 139 final VRZ trough concentrations, 105 (75.6%) were within the therapeutic target range, 7 were below 1.0 μg/mL and 27 were above 5.5 μg/mL.

### 3.3 VRZ TBIL-based dosing regimens and target attainment

151 initial TBIL values were available in the study cohort. The majority of recruited patients were prescribed a higher dose than their TBIL-based dose: 18 (11.9%) patients had the TBIL-based dose, 132 (87.4%) had a higher dose and 1 (0.7%) had a lower dose. Of these patients, 17/18, 122/132 and 1/1 initial VRZ trough concentrations were available. Initial target attainment was 76.5% (13/17), 66.4% (81/122) and 100% (1/1), respectively. Following TDM-guided dose adjustment, significantly more patients’ daily doses were equal to their TBIL-based dosing regimens (31 *versus* 18, *p* = 0.010).

### 3.4 Covariates that affecting VRZ trough concentration

**FIGURE 2:**
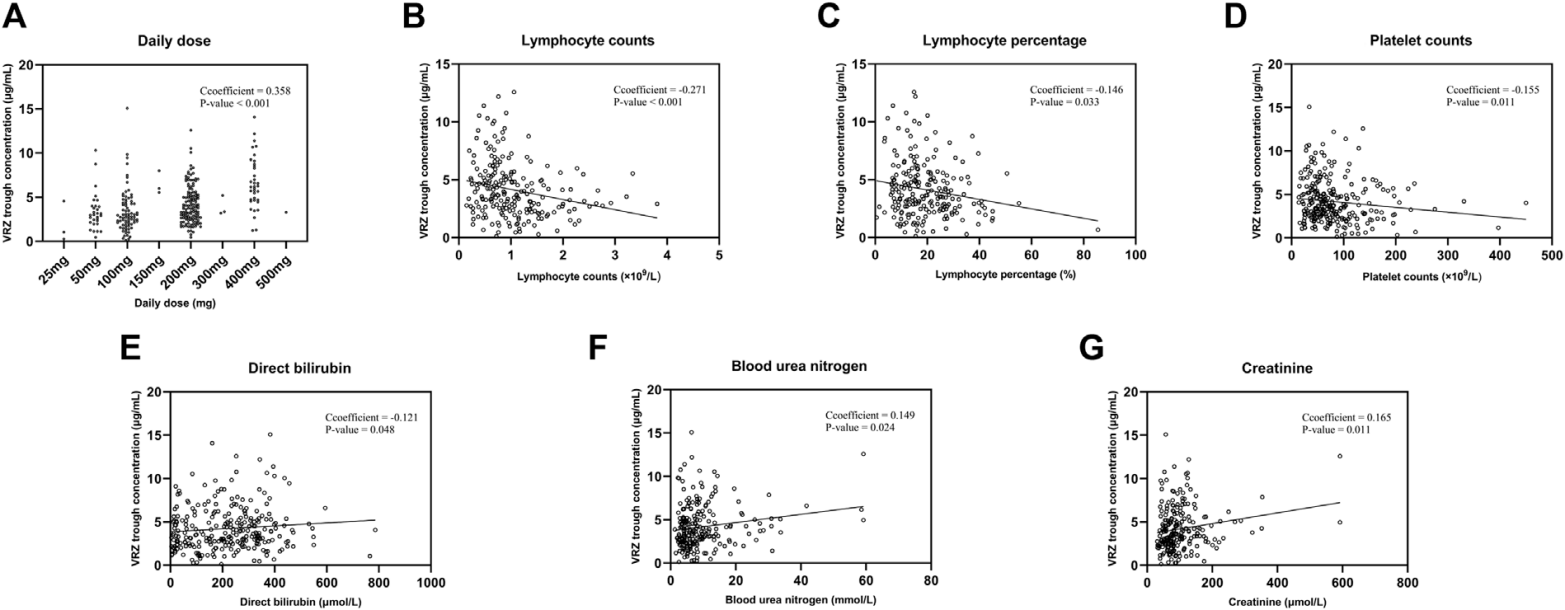
The correlation analysis between seven covariates and voriconazole trough concentrations. **(A)** Daily dose. **(B)** Lymphocyte counts. **(C)** Lymphocyte percentage. **(D)** Platelet counts. **(E)** Direct bilirubin. **(F)** Blood urea nitrogen. **(G)** Creatinine.

After Spearman correlation analysis, we screened seven factors, daily dose, direct bilirubin, lymphocyte counts and its percentage, blood urea nitrogen, platelet, creatinine, which affecting VRZ trough concentration significantly (Table 3). We performed multiple linear regression with stepwise method, the adjusted *R* ^2^ was 0.245 (Table 4). The correlation analysis between covariates and VRZ trough concentration are shown in Figure 2. To elaborate, every 1 mg increase in VRZ dose raises the trough concentration by 0.01 units. Meanwhile, a 1 μmol/L increase in creatinine results in a 0.007 unit rise in VRZ concentration. Besides, a 1 (109/L) increase in lymphocyte counts lowers the VRZ concentration by 0.576 units. And the multiple linear regression equation was as follows:

$$VRZ\;trough\;concentration\;\left(\mu g/mL\right)=2.263+0.01\times daily\;dose\;\left(mg\right)+0.007\times creatinine\;\left(\mu mol/L\right)-0.576\times lymphocyte\;counts\;\left(10^{9}/L\right)$$

**TABLE 3:** Spearman correlation analysis of factors associated with VRZ trough concentration.

Variable	Coefficient	*p*-value
Daily dose	0.358^a^	<0.001
RBC	−0.014	0.730
WBC	−0.089	0.176
PLT	−0.155^b^	0.011
HGB	0.021	0.732
LYM#	−0.271^a^	<0.001
LYM%	−0.146^b^	0.033
NEUT#	−0.050	0.476
NEUT%	0.110	0.112
ALT	0.044	0.472
AST	0.100	0.101
TBIL	0.118	0.054
DBIL	0.121^b^	0.048
ALB	0.073	0.235
ALP	−0.240	0.142
GGT	−0.082	0.626
CRP	0.157	0.166
PCT	0.166	0.110
BUN	0.149^b^	0.024
CREA	0.165^b^	0.011
UA	0.123	0.100
INR	0.079	0.226
PT	0.083	0.210
PT%	−0.027	0.828

^a^ *p*< 0.01 (2-tails).

^b^ *p*< 0.05 (2-tails).

**TABLE 4:** The multiple linear regression model about VRZ trough concentration^a^.

Variable	Unstandardized coefficients		Standardized coefficients	t	*p*-value	VIF^b^
	B	Std. Error				
Intercept	2.263	0.508		4.453	<0.001	
Daily dose	0.010	0.002	0.404	6.097	<0.001	1.016
CREA	0.007	0.002	0.211	3.175	0.002	1.019
LYM#	−0.576	0.249	−0.155	−2.316	0.022	1.035

^a^ *R* ^b^ = 0.258, adjusted *R*2 = 0.245, Durbin-Watson test value = 1.931, 287 VRZ, trough concentrations were analyzed.

^b^ VIF, variance inflation factor.

**FIGURE 3:**
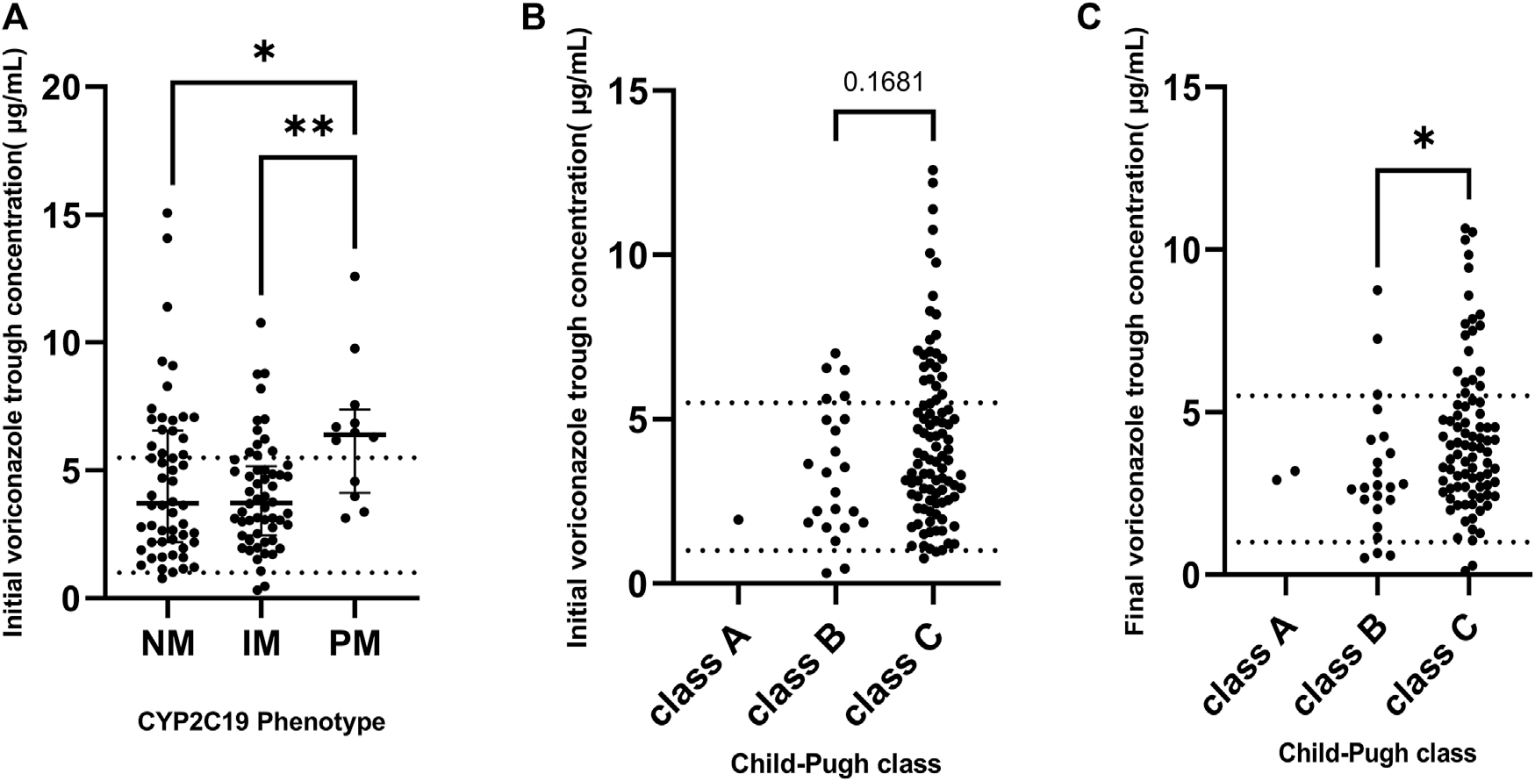
**(A)** The distribution of initial voriconazole trough concentrations grouped by CYP2C19 phenotype. **(B)** The distribution of initial voriconazole trough concentration grouped by baseline Child-Pugh class. **(C)** The distribution of final voriconazole trough concentration grouped by final Child-Pugh class. Dashed line: 1 and 5.5, respectively. *p < 0.05, **p < 0.01.

The distribution or target attainment of VRZ trough concentration for different CYP2C19 phenotypes, and different Child-Pugh class groups are shown in Figure 3. Significant differences were observed between the different CYP2C19 genotype groups and their initial VRZ trough concentrations. A pairwise comparison analysis is shown in Figure 3A according to phenotype. The initial VRZ trough concentrations of CYP2C19 PM were significantly higher than the NM (*p* = 0.0249) and IM (*p* = 0.0023), even higher than the upper limit of the therapeutic target range.

In addition, to further explore the impact factors of VRZ trough concentrations in relation to distinct CYP2C19 phenotypes, we carried out a subgroup analysis using Spearman correlation. The results demonstrated variability across these different subgroups (Supplementary Table S1–S3). For CYP2C19 NM group (Supplementary Table S1), both daily dose and albumin demonstrated a significant positive correlation with VRZ trough concentrations (*p* < 0.01), while lymphocyte counts showed a significant negative correlation (*p* < 0.05). In the case of CYP2C19 IM group (Supplementary Table S2), the daily dose again showed a significant positive correlation with VRZ trough concentrations (*p* < 0.01). However, in this group, lymphocyte counts, total bilirubin, and direct bilirubin were negatively correlated (*p* < 0.05). For CYP2C19 PM group (Supplementary Table S3), the daily dose continued to show a significant positive correlation (*p* < 0.01), and additional factors such as hemoglobin, neutrophil percentage, and uric acid also exhibited significant positive correlations with VRZ trough concentrations (*p* < 0.05).

### 3.5 Binary logistic regression of efficacy

**FIGURE 4:**
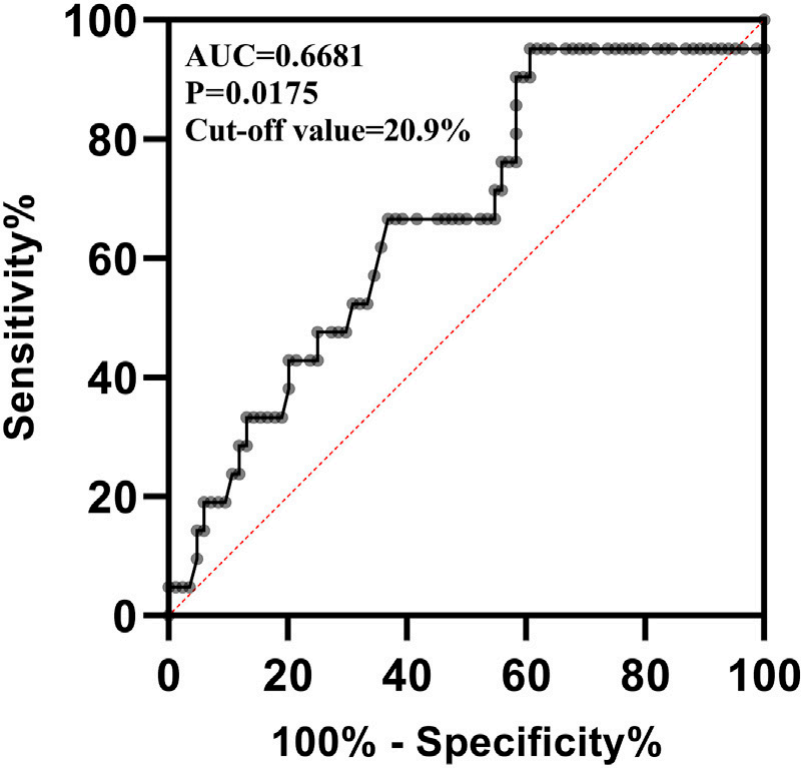
The receiver operating characteristic curve of lymphocyte percentage predicting the efficacy of voriconazole. AUC: Area Under the Curve.

VRZ was found to be efficacious in 122 out of 157 patients (77.7%). Baseline information was missing for 12 of the 157 patients included in the final dataset. Treatment interruption and discontinuation due to VRZ adverse events occurred in two patients, leaving 145 patients for inclusion in the binary logistic regression analysis. Of these patients, 112 (77.2%) were considered to have been effectively treated. Four covariates, including LYM%, neutrophils percentage (NEUT%), TBIL, and BUN were screened for further binary logistic regression analysis, with the *p*-value of the Hosmer-Lemeshow test being 0.930, indicating good predictive performance of the model. As shown in Table 5, LYM% exhibited a strong correlation with VRZ efficacy (*p* = 0.025) with a correlation coefficient of 0.129 and an odds ratio of 1.138 (95% CI: 1.016–1.273). The area under the receiver operating characteristic curve (AUC-ROC) was 0.668 (Figure 4), with a *p*-value of 0.0175, suggesting that LYM% could serve as a predictor of VRZ efficacy. Furthermore, a cut-off value of ≥20.9% was likely associated with higher VRZ therapeutic efficacy.

**TABLE 5:** The results of binary logistic regression of efficacy.

Covariant	B	*p*-value	OR^a^	95% CI^b^
LYM%	0.129	0.025	1.138	1.016–1.273
TBIL	−0.001	0.605	0.999	0.997–1.002
NEUT%	0.005	0.805	1.005	0.966–1.045
BUN	−0.006	0.798	0.994	0.951–1.039

^a^ OR, odds ratio.

^b^ CI, confidence interval.

By using the code written in sklearn package of Python to perform a simple machine learning, we obtained a ROC curve and AUC-ROC for predicting VRZ efficacy with LYM%. Likewise, the AUC-ROC output from Python is 0.6, which is close to the 0.668 calculated by SPSS. However, due to the low resolution of figure output by Python, the ROC curve output by Python has been added to the (Supplementary Figure S1).

## 4 Discussion

In accordance with previous studies (Dolton et al., 2012; Jin et al., 2016), subtherapeutic trough concentrations of VRZ have been associated with an increased risk of IFI. Conversely, supratherapeutic trough concentrations of VRZ have been linked to adverse events. Compared with other populations (Zhao et al., 2021b; Chen et al., 2022), the initial exposure of VRZ in patients with liver dysfunction appears to be higher. With decreased liver function, the metabolism and excretion of drug are slowed down and VRZ can easily accumulate (Verbeeck, 2008; Gonzalez et al., 2014). Consequently, it is critical for patients to achieve the therapeutic target range. This retrospective study sought to investigate the distribution and target attainment of VRZ trough concentration for different VRZ dose regimens, CYP2C19 polymorphisms, and Child-Pugh class groups. Moreover, we discovered that the major factor affecting VRZ efficacy in patients with liver dysfunction was LYM%, which was further validated by ROC analysis.

In our study, to explore the rationality of TBIL-based dosing regimens, we initially observed that after TDM (Therapeutic Drug Monitoring)-guided dose adjustment, a larger proportion of patients’ daily doses aligned with these regimens. Specifically, the number of patients on TBIL-based regimens increased from 18 to 31 post-adjustment (*p* = 0.010), highlighting the effectiveness of TBIL-based dosing in achieving target trough concentrations, particularly when compared to the empirical dosing approach. Further analysis revealed no significant difference in target attainment between patients whose dosing regimens matched the TBIL-based regimens and those whose regimens were higher. This observation led us to hypothesize that the CYP2C19 phenotype polymorphism, which results in different metabolic profiles, could be a contributing factor, leading to similar target attainment despite the variability in dosing regimens. Indeed, the results of Spearman correlation analyses of factors associated with Voriconazole (VRZ) trough concentrations across different CYP2C19 genotype groups support this hypothesis (Supplementary Table S1–S3). Overall, our investigation proposes that the CYP2C19 genotype could potentially affect the initial trough concentrations of VRZ, thereby implying a potential involvement in the development of initial dosing strategies. Nevertheless, the influence of the genotype on sustained trough levels was not observed, suggesting that other variables exert a more pronounced influence on the optimization of long-term VRZ concentrations. This discovery highlights the intricate nature of VRZ dosing and emphasizes the necessity of adopting a comprehensive approach to ensure effective therapy. Another aspect to consider is the limited sample size and the uncontrolled nature of our study. With only 17 patients administered the TBIL-based dosing regimens, the results, even when analyzed using Fisher’s exact test, may not fully capture the efficacy of the TBIL-based approach. Nevertheless, the increase in patients receiving TBIL-based regimens post-adjustment suggests that these regimens might be more appropriate than product information-based dosing for some patients.

Due to the limited numbers of CP-A patients (one in baseline and two in final) we did not analyze the difference between CP-A patients and CP-B/C patients. Nonetheless, both the target attainment and efficacy of CP-A patients were 100%, indicating that the dosing regimen (loading dose of 400 mg qd, maintenance dose of 200 mg qd) for CP-A patients was appropriate.

Various factors had been demonstrated to affect the VRZ trough concentration (Zhao et al., 2021b; Zhao et al., 2021; Tian et al., 2021). This study screened seven factors significantly affect the VRZ trough concentration and developed a multiple linear regression model. The final model could explain 24.5% of the variation of VRZ concentration. Specifically, creatinine was also one of the important predictors. Although voriconazole is predominantly metabolized in the liver, the correlation observed between voriconazole and renal function indicators in the multiple linear regression model may stem from a combination of factors. These include possible drug-drug interactions and the impact of various disease states (Brüggemann et al., 2009; Neofytos et al., 2012).

Moreover, this study also found that the initial VRZ trough concentration varied significantly across CYP2C19 genotypes, with the PM group showing higher levels than NM and IM groups. Despite this, there was no significant overall correlation between CYP2C19 genotype and VRZ trough concentration. This might be because the study considered all VRZ data points, not just initial ones. Initial measurements are likely more indicative of CYP2C19 genotype effects on metabolism, but over time, other elements like dosage and lymphocyte count could play a larger role, affecting VRZ levels in later measurements. Overall, the VRZ daily dose was positively correlated with its trough concentration significantly, which demonstrates that the necessity of reducing the VRZ dose in patients with liver dysfunction. Besides, some other factors that contribute to unpredictability of VRZ trough concentrations have been reported in published literature, such as inflammation (van Wanrooy et al., 2014; Encalada Ventura et al., 2016), polymorphism within drug transporters (Tilen et al., 2022), and the use of extracorporeal membrane oxygenation (Ye et al., 2022). In the analysis of relationship between CYP2C19 and initial VRZ trough concentration, our results were similar to previously studies (Kim et al., 2019; Zhang et al., 2021). Patients with CYP2C19 phenotype of PM exhibit the weak ability to metabolize the VRZ and their VRZ trough concentrations may be higher even when given the same dose. Moreover, due to the ethnic differences, the prevalence of the CYP2C19*17 allele in Asians is 4 times lower than White and African populations (Li-Wan-Po et al., 2010). Another study (Xie et al., 2001) reported that the frequency of CYP2C19*2 and *3 alleles in Chinese patients were far higher than the African and Caucasians.

In our study, the overall efficacy of VRZ treatment was 77.7%. The results of binary logistic regression of SPSS and machine learning indicate that as a predictor of the efficacy of VRZ, LYM% performed a good predictive effect. Binary logistic regression indicated that LYM% could predict the efficacy of VRZ, for every 1% increase in LYM%, the efficacy of VRZ will increase 1.138 times. Not coincidentally, the ROC curve showed a significantly higher probability of the efficacy of VRZ when LYM% was higher than 20.9%. Clinically, the normal reference range for LYM% is 20%–50%. Lymphocyte have the role of producing and transporting antibodies and defending against viral infections, its reduction is usually considered to be associated with immunodeficiency. Weiss E et al. (Weiss et al., 2020) found that one of the characteristics of patients with decompensated cirrhosis is lymphopenia. Other studies (Subesinghe et al., 2020; Yan and Wu, 2021) have demonstrated that lymphopenia predisposes to infection and is detrimental to the prognosis of infected patients. Besides, a decrease in LYM% may also due to an absolute value increase in neutrophil, resulting in a relatively low lymphocyte percentage, and the elevated counts of neutrophil are usually seen in various infections, for each unit increase of neutrophil-to-lymphocyte ratio, infection odds increases 1.29 times (Magalhães et al., 2021). Therefore, LYM% within the normal range facilitates the function of lymphocyte. Although we do not understand how the lymphocyte influence the VRZ *in vivo* currently, at least, we can assume that within the normal range, the higher the LYM%, the better for the patients. Whether it can be extrapolated to patients whose LYM% exceeds the upper limit of normal is unclear and requires further evaluation. Whereas, due to the limitation of sample size and the number of patients with inefficacy, the predictive power of the model predicting inefficacy is not as well as predicting efficacy. Therefore, it is necessary to further expand the sample size in the future to continuously improve our model.

In this study, we also found that NEUT counts (NEUT#) significantly related to the efficacy, but the correlation between NEUT# and NEUT% is strong, and the LYM% seems to be a better predictor in current study. Therefore, we did not take the NEUT# into the final analysis. In future research, we can keep an eye on the impact of these indicators on the concentration and efficacy of voriconazole.

We observed a robust association between C-reactive protein (CRP) and the efficacy of VRZ (*p* = 0.0003), which is consistent with previous reports (Chen et al., 2022; Jiang et al., 2022), demonstrating a significant impact of CRP on the pharmacokinetic profile of VRZ. Regrettably, only 44 CRP values were retrievable from the HIS, which were insufficient to provide a conclusive explanation for the efficacy observed in our final analysis. Therefore, a large prospective study is warranted to investigate the potential influence of CRP on the efficacy of VRZ in patients with liver dysfunction.

Our current study yielded several notable findings with implications for the use of VRZ in patients with liver dysfunction. Firstly, we investigated the rationality of TBIL-based dosing regimens and compared them with the recommended dose regimens. Additionally, we analyzed the distribution of VRZ trough concentrations across different Child-Pugh classes at both the initial and final stages of treatment. To the best of our knowledge, this is the first study to identify a correlation between LYM% and the effectiveness of VRZ. It is important to note some limitations of our study, which include its retrospective design and lack of clinical intervention. This may have resulted in missing data and confounding variables. Additionally, the sample size was limited, and there were few CYP2C19 UM and RM phenotypes in this study. Future studies should aim to elucidate the differences between these phenotypes and VRZ trough concentrations.

## 5 Conclusion

In conclusion, our study demonstrates that there is a substantial variation in VRZ trough concentration in patients with liver dysfunction. We identified seven covariates, including daily dose, direct bilirubin, LYM counts and percentage, platelet count, blood urea nitrogen, and creatinine, that significantly affect the VRZ trough concentration. Of note, LYM% is a robust predictor of the efficacy of VRZ, with each percentage increase of LYM% resulting in a 1.138 times increase in efficacy. Although TBIL-based dosing regimens appear to be appropriate, a large multicenter prospective study is needed to further confirm this finding.

## Data Availability

The raw data supporting the conclusion of this article will be made available by the authors, without undue reservation.
