# Supplementary material for: Enhancing voriconazole therapy in liver dysfunction: exploring administration schemes and predictive factors for trough concentration and efficacy
Source: Front Pharmacol. 2024 Jan 4;14:1323755. doi: 10.3389/fphar.2023.1323755 (PMC10794455; doi:10.3389/fphar.2023.1323755)
Supplement: Supplementary file 1 [file DataSheet1.docx]

Supplementary Material

## 1 Supplementary Table

**Supplementary Table 1.** Spearman correlation analysis of factors associated with VRZ trough concentration in CYP2C19 normal metabolizers

| Variable | Coefficient | p-value |
| --- | --- | --- |
| Daily dose | 0.320^**^ | 0.001 |
| RBC | -0.029 | 0.795 |
| WBC | -0.049 | 0.650 |
| PLT | -0.131 | 0.187 |
| HGB | 0.077 | 0.441 |
| LYM# | -0.258^*^ | 0.020 |
| LYM% | 0.190 | 0.087 |
| NEUT# | 0.018 | 0.874 |
| NEUT% | 0.130 | 0.250 |
| ALT | 0.095 | 0.346 |
| AST | 0.168 | 0.094 |
| TBIL | 0.012 | 0.909 |
| DBIL | 0.045 | 0.654 |
| ALB | 0.295^**^ | 0.003 |
| ALP | -0.270 | 0.350 |
| GGT | -0.160 | 0.584 |
| CRP | 0.206 | 0.208 |
| PCT | 0.215 | 0.161 |
| BUN | 0.189 | 0.073 |
| CREA | 0.188 | 0.075 |
| UA | 0.123 | 0.318 |
| INR | -0.074 | 0.499 |
| PT | -0.049 | 0.660 |
| PT% | 0.088 | 0.634 |

^*^ p< 0.05 (2-tails)

^**^ p< 0.01 (2-tails)

**Supplementary Table 2.** Spearman correlation analysis of factors associated with VRZ trough concentration in CYP2C19 intermediate metabolizers

| Variable | Coefficient | p-value |
| --- | --- | --- |
| Daily dose | 0.341^**^ | <0.001 |
| RBC | -0.168 | 0.165 |
| WBC | -0.018 | 0.873 |
| PLT | -0.139 | 0.177 |
| HGB | -0.085 | 0.407 |
| LYM# | -0.332^**^ | 0.006 |
| LYM% | -0.156 | 0.205 |
| NEUT# | -0.038 | 0.760 |
| NEUT% | 0.084 | 0.498 |
| ALT | -0.077 | 0.444 |
| AST | 0.045 | 0.652 |
| TBIL | 0.225^*^ | 0.026 |
| DBIL | 0.237^*^ | 0.019 |
| ALB | -0.157 | 0.123 |
| ALP | -0.175 | 0.587 |
| GGT | -0.045 | 0.894 |
| CRP | 0.068 | 0.737 |
| PCT | 0.038 | 0.849 |
| BUN | 0.076 | 0.076 |
| CREA | 0.111 | 0.308 |
| UA | -0.003 | 0.979 |
| INR | 0.168 | 0.117 |
| PT | 0.159 | 0.141 |
| PT% | -0.050 | 0.799 |

^*^ p< 0.05 (2-tails)

^**^ p< 0.01 (2-tails)

**Supplementary Table 3.** Spearman correlation analysis of factors associated with VRZ trough concentration in CYP2C19 poor metabolizers

| Variable | Coefficient | p-value |
| --- | --- | --- |
| Daily dose | 0.533^**^ | 0.007 |
| RBC | 0.362 | 0.106 |
| WBC | 0.145 | 0.529 |
| PLT | -0.040 | 0.854 |
| HGB | 0.474^*^ | 0.022 |
| LYM# | -0.129 | 0.610 |
| LYM% | -0.342 | 0.165 |
| NEUT# | 0.187 | 0.458 |
| NEUT% | 0.515^*^ | 0.029 |
| ALT | 0.225 | 0.290 |
| AST | 0.252 | 0.235 |
| TBIL | -0.162 | 0.450 |
| DBIL | -0.079 | 0.713 |
| ALB | -0.161 | 0.453 |
| ALP | -0.500 | 0.391 |
| GGT | -0.100 | 0.873 |
| BUN | 0.276 | 0.226 |
| CREA | 0.173 | 0.452 |
| UA | 0.631^*^ | 0.016 |
| INR | 0.216 | 0.375 |
| PT | 0.216 | 0.375 |
| PT% | -0.738 | 0.155 |

^*^ p< 0.05 (2-tails)

^**^ p< 0.01 (2-tails)

## 2 Supplementary Figure


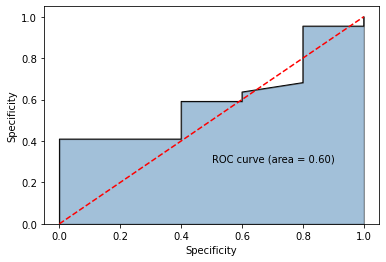


**Supplementary Figure 1.** The receiver operating characteristic curve output by Python of lymphocyte percentage predicting the efficacy of voriconazole.
